# Two‐dimensional echocardiographic right heart ratios for assessment of right heart size in dogs: Reference intervals and reproducibility

**DOI:** 10.1111/jvim.17159

**Published:** 2024-09-30

**Authors:** Jacqueline N. Sankisov, Lance C. Visser, Kate E. Davis, June A. Boon, Evan S. Ross, Abigail C. Laws

**Affiliations:** ^1^ Department of Clinical Sciences, College of Veterinary Medicine & Biomedical Sciences Colorado State University Fort Collins Colorado USA

**Keywords:** canine, echocardiography, reference range, right atrium, right ventricle

## Abstract

**Background:**

Reference intervals for simple body weight‐independent measurements of right heart size and function are limited.

**Objectives:**

Generate reference intervals for measurements of right heart size indexed to the long‐axis aortic valve diameter (AoD) or corresponding left heart structure (right heart ratios) and describe the reproducibility of these indices.

**Animals:**

Ninety healthy adult dogs of variable body weight.

**Methods:**

Prospective study. All dogs underwent an echocardiogram performed by the same operator. Numerous linear 2‐dimensional measurements of right heart size and function from different imaging planes were performed. Eight dogs underwent repeated echocardiograms by the same operator on 3 different days, and 3 different operators performed repeated echocardiograms on the same day. Reference intervals were generated using the Clinical Laboratory Standards Institute method. Reproducibility was quantitated using coefficients of variation (CVs) and reproducibility coefficients.

**Results:**

Reference intervals for right heart ratios were generated and allow simple assessments of right heart size and function that do not require a scaling exponent or body weight table. Right heart ratios did not show clinically relevant associations with body weight. All CVs were <22.6%. In general, CVs for right heart measurements indexed to AoD were lower compared with right heart measurements indexed to the corresponding left heart structure.

**Conclusions and Clinical Importance:**

Reference intervals for simple body weight‐independent right heart ratios are available to help detect abnormalities of right heart size and function. Reproducibility coefficients might be useful to help identify meaningful changes in right heart size during serial evaluations.

**Abbreviations**
AoD: aortic valve diameter in long‐axis
Ap4Ch: apical 4‐chamber view
CV: coefficient of variation
EId: eccentricity index at end‐diastole
LA: left atrial
LAD: left atrial dimension
LVD1: latero‐lateral left ventricular dimension
LVD: cranio‐caudal left ventricular dimension
LVDbase: basilar left ventricular dimension
LVWT: left ventricular wall thickness
PVdia: pulmonary valve diameter
RAD: right atrial dimension
RPLx: right parasternal long‐axis view
RPSx: right parasternal short‐axis view
RV: right ventricular
RVD: right ventricular dimension
RVDbase: basilar right ventricular dimension
RVDmid: midchamber right ventricular dimension
RVTW: right ventricular wall thickness
TAPSE: tricuspid annular plane systolic excursion

## 1 INTRODUCTION

The importance of quantitative echocardiographic assessment of right heart size and function is becoming increasingly apparent in dogs affected with a variety of cardiovascular diseases, including myxomatous mitral valve disease,^1^, ^2^, ^3^, ^4^ cardiomyopathies,^5^, ^6^, ^7^ pulmonary stenosis,^8^, ^9^, ^10^ and precapillary pulmonary hypertension.^11^, ^12^, ^13^, ^14^ Despite this utility, routine quantitative assessment of right heart size is uncommon. Most sonographers seem to rely on subjective assessment and compare the size of a right heart structure to the corresponding left heart structure.

Routine measurements of right heart structures are uncommon, likely because they are more challenging compared with the left heart. Also, normative data for simple, body weight‐independent measurements of right heart size are limited.^15^ Most prospective studies designed to generate reference intervals in a standardized manner have focused on indices of right ventricular (RV) systolic function and to a lesser extent on right atrial or RV size and wall thickness.^16^, ^17^, ^18^, ^19^, ^20^

In dogs, several strategies for normalizing cardiac measurements to body size have been used for the assessment of the left heart size. These include normalizing to body weight using nonlinear regression or allometric scaling^21^, ^22^, ^23^, ^24^ and indexing linear measurements to an internal control such as the diameter of the aorta. Indexing measurements to the aortic diameter from a right parasternal short‐axis (RPSx) basilar view^25^, ^26^, ^27^, ^28^ or long‐axis left ventricular outflow tract view^23^, ^29^ has been proposed. The latter avoids some of the challenges associated with short‐axis measurement of the aortic root, which include inconsistencies in the timing of the measurement^30^, ^31^ and defining the path of the measurement relative to the valve sinus. The long‐axis aortic valve diameter (AoD) is measured during early to midsystole (largest diameter) between the hinge points of the maximally opened aortic valve cusps and does not include a sinus of Valsalva.^23^, ^29^, ^32^ When comparing simple aortic ratios for left atrial (LA) size assessment, the long‐axis LA/AoD method has been shown to exhibit superior reproducibility compared with the short‐axis aortic root LA/Ao method in healthy dogs and dogs with myxomatous mitral valve disease.^23^, ^33^ However, specific comparisons of the reproducibility of AoD versus short‐axis aortic root measurements are not reported in these studies. The superiority of 1 aortic measurement method versus the other has not been determined. Thus, the method of indexing to the aorta (long‐ vs short‐axis) is largely personal preference.

Studies proposing body size‐independent reference intervals of quantitative measurements of right heart size and function currently are limited to normalizing to body weight^13^, ^14^, ^15^, ^16^, ^17^, ^19^, ^20^, ^34^ or indexing to short‐axis aortic diameter.^18^ Right heart measurements indexed to the long‐axis aortic diameter or corresponding left heart structure, henceforth referred to as right heart ratios, might offer some advantages. These include situations in which body weight is poorly representative of body size, such as obesity or cachexia. Right heart ratios also might offer advantages during focused echocardiography in cardiac emergency settings in which body weight or body weight‐based echocardiographic measurement charts are unavailable.

Our aims were to generate simple, body size‐independent reference intervals for right heart ratios that were acquired prospectively during a systematic examination of healthy dogs and to describe the reproducibility of these measurements. A secondary aim was to evaluate the agreement between analogous right heart measurements from different echocardiographic imaging planes.

## 2 ANIMALS, MATERIALS, AND METHODS

Study methods were approved by the Institutional Animal Care and Use Committee at Colorado State University (protocol no. 4429). Dog owners provided written consent before enrolling their dogs.

### 2.1 Animals

A convenience sample of healthy privately owned dogs was recruited from the community associated with Colorado State University College of Veterinary Medicine and Biomedical Sciences over a 3‐month period. Dogs were eligible for inclusion if they were at least 1 year of age, healthy (no current or recent illness and not previously diagnosed with heart disease), not taking medications that affect the cardiovascular system, and compliant and able to lie still for an echocardiographic examination. The dogs were healthy based on patient history, physical examination, and a thorough screening echocardiographic examination performed by a cardiologist (LCV). This examination included standard imaging planes,^35^ and a careful evaluation for cardiac abnormalities including subjective abnormalities of cardiac chamber size and function, and color and spectral Doppler interrogation of all cardiac valves and septa. Dogs were excluded if they had a ≥3/6 heart murmur, they would not lie still for most of the echocardiographic examination, or if any cardiac abnormalities or nonsinus arrhythmias (on simultaneous ECG) were found by echocardiography. Trace valvular regurgitation (subjective assessment) based on color Doppler imaging of each valve was allowed provided no structural abnormalities were found. Dogs were allowed to receive anxiolytic drugs (ie, trazodone, gabapentin PO) before examination if their owners thought it would be to their dogs' benefit. Parenteral sedatives were not allowed. All dogs were weighed on the same digital floor scale at the time of their first echocardiographic examination.

### 2.2 Study design

Each dog underwent an echocardiographic examination by the same operator (LCV). Eight dogs of variable size were selected to undergo additional examinations to evaluate reproducibility of the echocardiographic measurements (ie, variability of the same measurement made on the same subject under the changing conditions of day or operator). These dogs were selected based on owner availability and willingness to participate. To evaluate interoperator reproducibility, dogs underwent repeated echocardiograms by 3 operators, a cardiology resident (ESR), an experienced sonographer (JAB), and a cardiologist (LCV), on the same day. To evaluate intraoperator reproducibility, the same operator (LCV) performed repeated echocardiograms on 3 different days within 1 week. Thus, each of the 8 dogs underwent 5 echocardiographic examinations for the reproducibility portion of the study. Each operator performed his or her own measurements. They were masked to earlier measurements and measurements of the other operators at the time of measurement.

### 2.3 Echocardiographic examination

Echocardiographic examinations were performed using the same ultrasound unit (Philips EPIQ 7C, Philips Healthcare, Andover, Massachusetts) equipped with several cardiac transducers and simultaneous ECG. Choice of carrier frequency and techniques to optimize image quality was performed at the discretion of the operator. Dogs were gently manually restrained in right and left lateral recumbency. Data were captured digitally for measurements at an off‐cart workstation (Syngo Dynamic Workplace, Siemens Medical Solutions, Inc, Malvern, Pennsylvania). Images and cine loops used for study purposes were performed using conventional imaging planes, unless specified.^35^

A single trained investigator (JNS, a cardiology research intern) performed all measurements that were not involved in the reproducibility study. To evaluate training of this investigator and for quality control purposes to assess measurement variability and consistency, 8 dogs were randomly selected and remeasured on 2 separate days and measured by 2 additional study investigators (LCV and ACL). At the time of measurement, investigators were masked to each other's measurements and their earlier measurements. For all measurements, each reported variable consisted of an average of 3, usually consecutive, cardiac cycles.

All measurements were performed using 2‐dimensional echocardiography unless specified. For measurements in which atrioventricular valves were visible, end‐diastole was defined as the frame coincident with valve closure. If atrioventricular valves could not be visualized (RPSx measurements), end‐diastole was defined at the peak of the R wave on the ECG or the largest chamber dimension. End‐systole was defined as the frame just before atrioventricular valve opening or the smallest chamber dimension. All internal cardiac chamber measurements were performed from inner edge‐to‐inner edge at the blood‐tissue interface. All internal right heart chamber measurements were performed first and any corresponding left heart chamber dimension (see below) was performed second and continued approximately along the same line/trajectory as the right heart measurement. This was not necessarily the case for wall thickness measurements, which were confined to the thickest measurement within a region (basilar 1/3 for long‐axis, central 1/3 for short‐axis; see below). Wall thickness measurements were performed at end‐diastole from the inner edge of the endocardium (blood‐tissue interface) to the outer edge epicardium, excluding the pericardium.

**FIGURE 1:**
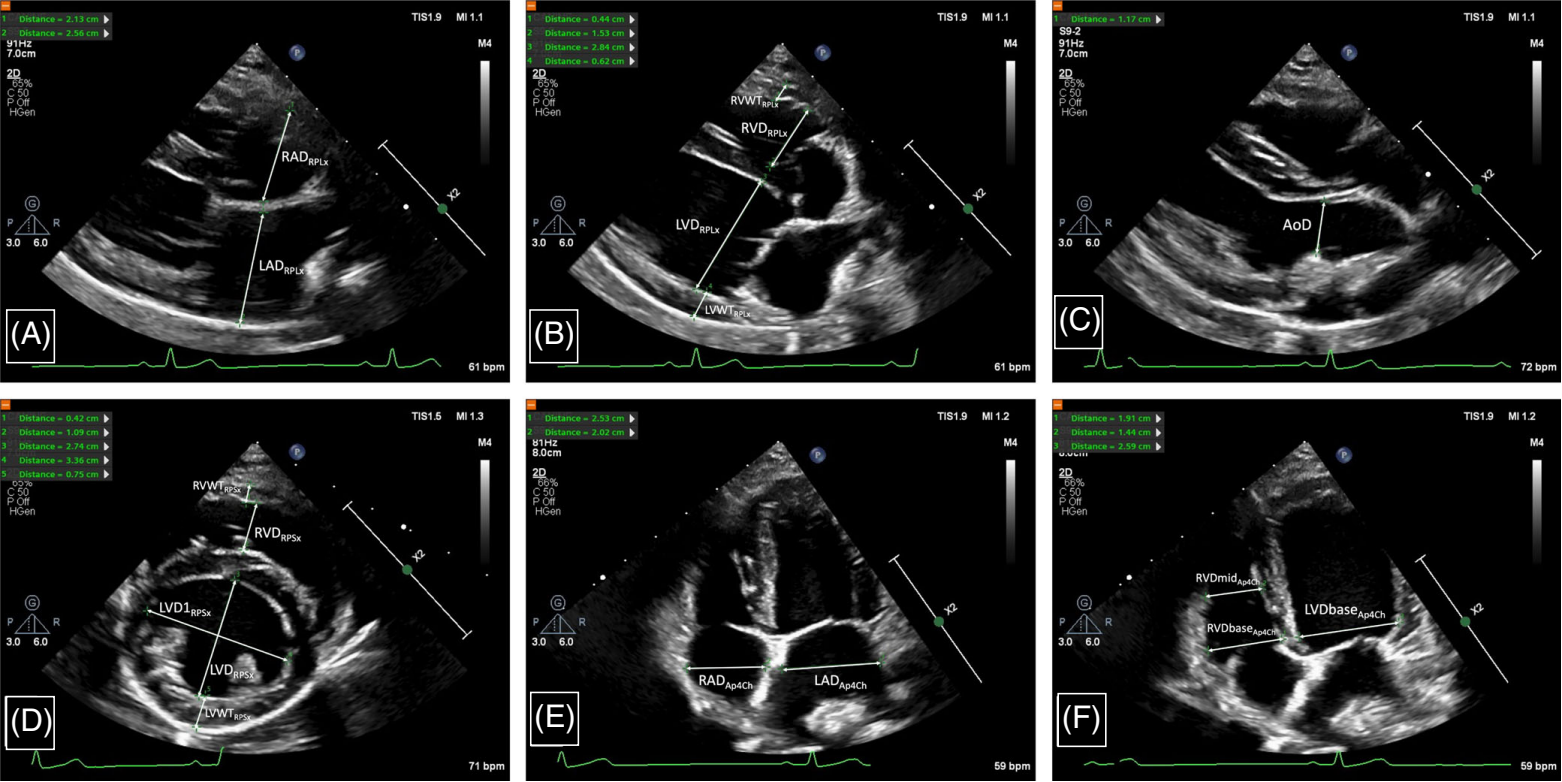
Example measurements of right atrial dimension (RAD) and left atrial dimension (LAD) from a right parasternal long‐axis (RPLx) 4‐chamber view at end‐systole (A), right ventricular wall thickness (RVWT), right ventricular dimension (RVD), left ventricular dimension (LVD), and left ventricular wall thickness (LVWT) at end‐diastole (B), aortic valve diameter (AoD) in early to midsystole measured between the hinge points of the maximally opened aortic valve cusps from a right parasternal long‐axis view optimized for the left ventricular outflow tract and ascending aorta (C), RVWT, RVD, cranio‐caudal LVD, latero‐lateral left ventricular dimension (LVD1), and LVWT from a right parasternal short‐axis view (RPSx) optimized for the high papillary muscles/chordal attachments at end‐diastole (D), RAD and LAD from a left apical 4‐chamber view (Ap4Ch) with the beam centered on or just to the left of the interventricular septum at end‐systole (E), and, in the basilar one third of the chamber, right ventricular dimension (RVDbase) and left ventricular dimension (LVDbase), and right ventricular dimension at the midchamber level (RVDmid) from a left Ap4Ch view at end‐diastole (F). See the text for further details on performing these measurements.

A right parasternal long‐axis 4‐chamber view (RPLx) optimized for maximizing LA size and the left ventricular inflow tract was used for the study. Specifically, the heart was slightly tilted by pointing the crystals cranially and dorsally and moving the probe slightly ventral and sometimes caudally to bring the right atrium into the sector while maximizing LA size and maintaining visualization of the atrial septum, fossa ovalis, and entrance of the right pulmonary vein into LA. From this view at end‐systole, right atrial dimension (RAD_RPLx_) was measured midchamber and approximately parallel to the tricuspid annulus from the level of the fossa ovalis to the right atrial free wall in the near field (Figure 1A). An analogous measurement of the left atrial dimension (LAD_RPLx_) was performed as previously described in the introduction (Figure 1A).^23^, ^29^ From the same RPLx view but at end‐diastole, right ventricular wall thickness (RVWT_RPLx_), basilar right ventricular dimension (RVDbase_RPLx_), basilar left ventricular dimension (LVDbase_RPLx_), and left ventricular free wall thickness (LVWT_RPLx_) were measured (Figure 1B). For RVWT_RPLx_, maximum wall thickness was measured within the basal one third of the wall. The RVDbase_RPLx_ was measured as the largest dimension approximately parallel to the tricuspid annulus in the basal one third of the RV chamber. The LVDbase_RPLx_ and LVWT_RPLx_ measurements were made in an equivalent fashion. The AoD was measured from a RPLx optimized for the left ventricular outflow tract and ascending aorta as previously described (Figure 1C).

From the RPSx optimized for the high papillary muscles/chordal attachments, RVWT_RPSx_, RV dimension (RVD_RPSx_), cranio‐caudal left ventricular dimension (LVD_RPSx_), the widest latero‐lateral left ventricular dimension (LVD1_RPSx_) that was perpendicular to LVD_RPSx_,^36^ and LVWT_RPSx_ were measured at end‐diastole, and with the exception of LVD1_RPSx_, within the central one third of the sector at their largest dimension (Figure 1D). Care was taken to avoid inclusion of papillary muscles within a measurement, especially for RVD_RPSx_ (ie, wall thickness measurements did not include papillary muscles). Left ventricular eccentricity index at end‐diastole (EId) was calculated as $\mathrm{LVD}1_{\text{RPSx}}÷{\mathrm{LVD}}_{\text{RPSx}}$.^36^

From the RPSx basilar view optimized for the right ventricular outflow tract, pulmonary valve, and pulmonary arteries, pulmonary valve diameter (PVdia_RPSx_) was measured between the valve hinge points (avoiding the valve sinus) in early to midsystole when the cusps were maximally opened.

A left apical 4‐chamber view (Ap4Ch) with the ultrasound beam centered on or just to the left of the ventricular septum and optimized for the right atrium and RV inlet was used for the study. Care was taken to elongate the RV as much as possible and to avoid visualization of the aortic valve and caudal vena cava. From this view at end‐systole, RAD_Ap4Ch_ and LAD_Ap4Ch_ were measured using an analogous method to the RPLx view (Figure 1E). At end‐diastole, RV dimensions were measured at the maximum dimension parallel to the tricuspid annulus in the basal one third of the right ventricular chamber (RVDbase_Ap4Ch_) and at the midchamber level (RVDmid_Ap4Ch_; Figure 1F).^37^ A left ventricular dimension in the basilar one third of the left ventricular chamber (LVDbase_Ap4Ch_) equivalent to RVDbase_Ap4Ch_ also was measured (Figure 1F). Tricuspid annular plane systolic excursion (TAPSE) was measured using M‐mode.^17^, ^20^ Care was taken to avoid incorporating tricuspid valve motion within the M‐line. An initial attempt was made to measure maximum end‐diastolic RVWT at the midchamber level from this Ap4Ch view, but we chose to stop collecting this measurement because of inability to visualize the RV epicardial‐pericardial border consistently.

For comparison, 2 measurements (RAD_Ap4Ch_ and RVDmid_Ap4Ch_) from all dogs were compared with equivalent measurements from previously published body weight‐based prediction intervals for RA and RV chamber size.^15^ A RAD_Ap4Ch_ >9.02 mm/kg^0.39^ and RVDmid_Ap4Ch_ >9.35 mm/kg^0.33^ were considered abnormal (ie, beyond the upper 97.5 percentile). Values outside these prediction intervals did not prevent enrollment into the study.

### 2.4 Statistical analyses

Statistical analyses were performed using commercially available software (MedCalc Statistical Software, MedCalc Software bvba, Ostend, Belgium). All echocardiographic indices were visually inspected using a box‐and‐whisker plot and evaluated for outliers using Tukey's method. Statistical outliers were examined and only considered for removal if an obvious measurement error was suspected. Ninety‐five percent reference intervals were calculated using a robust method as recommended by the Clinical Laboratory Standards Institute guidelines for sample sizes <120 subjects.^38^ Ninety percent confidence intervals were determined for each reference limit using a percentile interval method bootstrapping technique. After verifying that assumptions were met, Bland‐Altman analyses with 95% limits of agreement (plotting differences as a percentage) were generated to compare equivalent right heart size measurements from different echocardiographic imaging planes. Paired *t* test and simple linear regression were used to decide if fixed and proportional biases were apparent, respectively. Reproducibility of repeated echocardiographic measurements was quantitated using coefficients of variation (CVs) and 95% reproducibility coefficients (95% RC). These were derived from the within‐subject standard deviation (wSD).^39^, ^40^ The wSD was calculated as the square root of the within‐subject variance (mean square error), which was determined from a 1‐way analysis of variance (ANOVA) with dogs as the grouping variable. The CV was calculated as $\left(\mathrm{wSD}÷\text{overall mean}\right)\times 100$, and 95% RC was calculated as 1.96×√2×wSD.^40^ Coefficients of variation also were generated to assess the measurement variability of the investigator that performed all echocardiographic measurements (JNS). To verify the right heart ratios eliminated a clinically relevant relationship to body weight, Spearman's rank correlation coefficients (rho) were generated between each right heart ratio and body weight. Only rho >0.4 were considered to be clinically relevant.^23^ Normality testing was performed using the Shapiro‐Wilk test. *P* < .05 was used to denote statistical significance.

## 3 RESULTS

Ninety‐two dogs were recruited and underwent an examination. One dog was excluded because it had an uncooperative temperament and 1 because it was diagnosed with mild myxomatous mitral valve disease. Thus, 90 dogs were used to generate the normative data. Thirty‐seven dogs were female and 53 were male. Median (minimum, maximum) body weight and age were 25 (2.3, 55.9) kg and 6.2 (1.0, 15.4) years, respectively. The study sample had a variety of large and small mixed breeds (n = 54) and purebred dogs (n = 36). There were 5 Labrador retrievers, 4 golden retrievers, 4 German shorthaired pointers, 2 standard poodles, 2 shih tzus, 2 German shepherd dogs, 2 Welsh springer spaniels, 2 Doberman pinschers, and 1 each of mudi, Bernese mountain dog, saluki, Rottweiler, Chihuahua, miniature poodle, English bulldog, beagle, miniature Australian shepherd, English Labrador, Belgian Malinois, cane corso, and dachshund.

The 8 dogs examined for the reproducibility portion of the study consisted of a 7‐year‐old male 23 kg mixed breed dog, a 2‐year‐old female 25 kg mixed breed dog, a 4‐year‐old male 37 kg mixed breed dog, a 5‐year‐old female 46 kg mixed breed dog, a 1‐year‐old male 51 kg Cane Corso, a 9‐year‐old male 32 kg Labrador retriever, an 11‐year‐old 4 kg male mixed breed dog, and a 9‐year‐old female 31 kg mixed breed dog.

Except for RVWT_Ap4Ch_, all right heart ratios could be acquired and measured in each dog. No obvious measurement errors were found on the measurements determined to be statistical outliers. Thus, no measurements were excluded from the data analysis.

Proposed reference intervals for the echocardiographic right heart ratios are summarized in Table 1. For RAD_Ap4Ch_, 5 dogs would be considered abnormal (>9.02 mm/kg^0.39^) compared with body weight‐based prediction intervals for which values were 10.17, 9.57, 9.20, 9.20, and 9.55 mm/kg^0.39^. For RVDmid_Ap4Ch_, 2 dogs would be considered abnormal (>9.35 mm/kg^0.33^) for which values were 10.54 and 11.29 mm/kg^0.33^.

**TABLE 1:** Proposed reference intervals for measurement of right heart size and function indexed to the long‐axis aortic diameter or corresponding left heart structure (right heart ratios) from 90 healthy dogs.

Echocardiographic indices (indexed)	Median	Minimum‐maximum	95% reference interval
			2.5 centile (90% CI) to 97.5 centile (90% CI)
Right atrial size			
RAD_RPLx_/AoD	1.51	0.97‐2.07	1.08 (1.01, 1.14) to 1.95 (1.89, 2.01)
RAD_AP4Ch_/AoD	1.41	0.79‐2.02	0.89 (0.82, 0.97) to 1.90 (1.83, 1.98)
RAD_RPLx_/LAD_RPLx_	0.74	0.51‐0.99	0.53 (0.50, 0.56) to 0.95 (0.92, 0.98)
RAD_AP4Ch_/LAD_AP4Ch_	0.80	0.46‐1.34	0.51 (0.46, 0.56) to 1.07 (1.02, 1.12)
Right ventricular chamber size			
RVDbase_RPLx_/AoD	1.10	0.72‐1.62	0.67 (0.61, 0.73) to 1.57 (1.50, 1.63)
RVD_RPSx_/AoD	0.82	0.38‐1.47	0.38 (0.32, 0.45) to 1.23 (1.16, 1.30)
RVDbase_AP4Ch_/AoD	1.31	0.81‐1.94	0.83 (0.77, 0.90) to 1.76 (1.69, 1.83)
RVDmid_AP4Ch_/AoD	1.15	0.75‐1.71	0.70 (0.65, 0.77) to 1.64 (1.56, 1.71)
RVDbase_RPLx_/LVDbase_RPLx_	0.52	0.29‐0.74	0.33 (0.31, 0.36) to 0.68 (0.65, 0.71)
RVD_RPSx_/LVD_RPSx_	0.36	0.21‐0.64	0.19 (0.16, 0.21) to 0.54 (0.51, 0.57)
RVDbase_AP4Ch_/LVDbase_AP4Ch_	0.56	0.40‐0.82	0.37 (0.34, 0.39) to 0.76 (0.72, 0.79)
Right ventricular wall thickness			
RVWT_RPLx_/AoD	0.37	0.24‐0.58	0.25 (0.23, 0.27) to 0.49 (0.47, 0.52)
RVWT_RPSx_/AoD	0.35	0.23‐0.54	0.23 (0.22, 0.25) to 0.48 (0.46, 0.50)
RVWT_RPLx_/LVWT_RPLx_	0.62	0.44‐0.88	0.45 (0.43, 0.48) to 0.78 (0.76, 0.81)
RVWT_RPSx_/LVWT_RPSx_	0.60	0.45‐0.88	0.43 (0.40, 0.46) to 0.77 (0.74, 0.80)
Other			
PVdia_RPSx_/AoD	1.00	0.76‐1.53	0.74 (0.70, 0.80) to 1.29 (1.23, 1.34)
EId	1.04	0.81‐1.32	0.83 (0.80, 0.87) to 1.22 (1.19, 1.26)
TAPSE/AoD	0.80	0.47‐1.20	0.48 (0.43, 0.53) to 1.18 (1.12, 1.22)

Abbreviations: AoD, aortic valve diameter in long‐axis; Ap4Ch, apical 4‐chamber view; CI, confidence interval; EId, eccentricity index at end‐diastole; LAD, left atrial dimension; LVD, cranio‐caudal left ventricular dimension; LVDbase, basilar left ventricular dimension; LVWT, left ventricular wall thickness; PVdia, pulmonary valve diameter; RAD, right atrial dimension; RPLx, right parasternal long‐axis view; RPSx, right parasternal short‐axis view; RVD, right ventricular dimension; RVDbase, basilar right ventricular dimension; RVDmid, midchamber right ventricular dimension; RVTW, right ventricular wall thickness; TAPSE, tricuspid annular plane systolic excursion.

No clinically relevant correlations between right heart ratios and body weight were found. Three ratios yielded weak significant associations with body weight: RVWT_RPLx_/AoD (rho = −0.3; *P* = .001), RVWT_RPSx_/AoD (rho = −0.34; *P* = .001), and TAPSE/AoD (rho = −0.26; *P* = .02).

**FIGURE 2:**
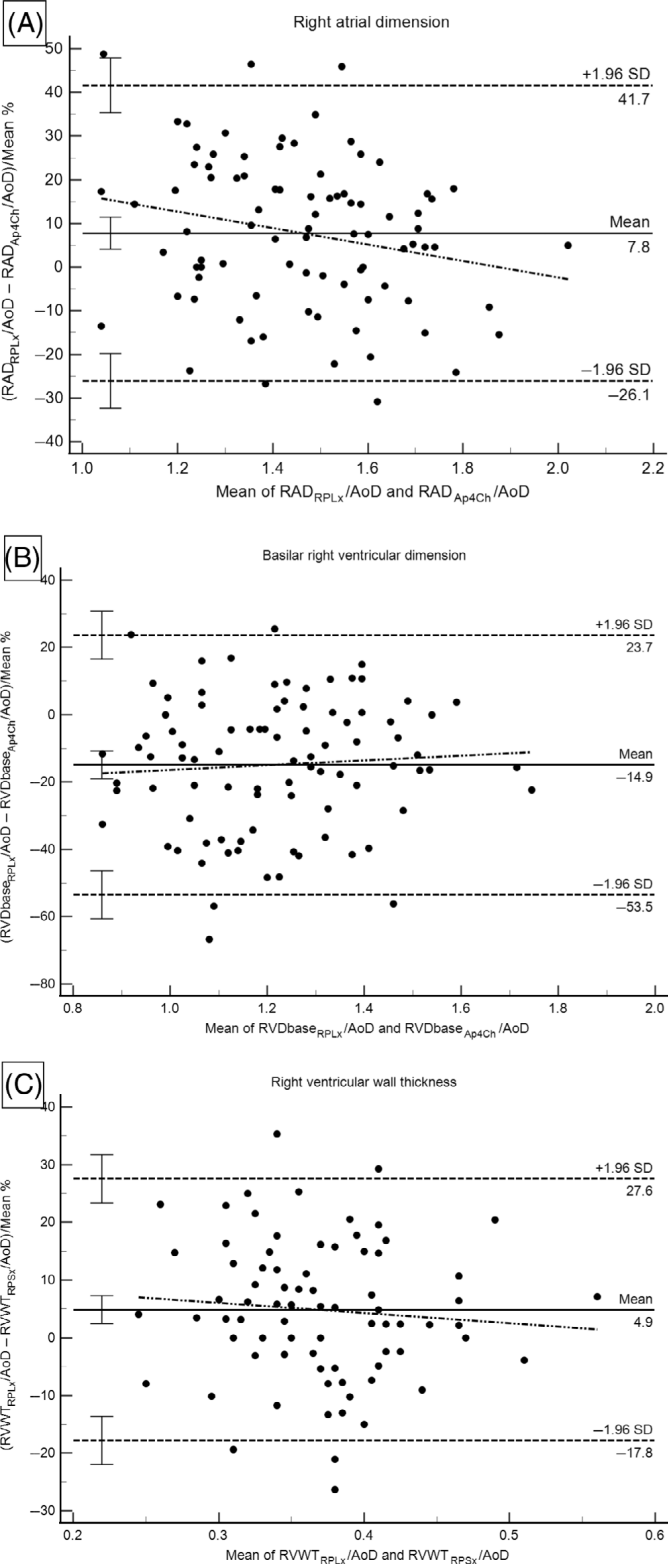
Results of the Bland‐Altman plot analyses evaluating agreement for equivalent right heart size measurements from different echocardiographic views for right atrial dimension (A), basilar right ventricular dimension (B), and right ventricular wall thickness (C). Each scatter diagram plots the differences as a percent of the mean (*y*‐axis) against the mean (*x*‐axis) of the two methods and show the 95% limits of agreement (dashed lines), each with 95% confidence intervals, mean difference as % (solid line) with a 95% confidence interval, and linear regression line (dotted and dashed line). AoD, aortic valve diameter from a right parasternal long‐axis view; Ap4Ch, apical 4‐chamber view RAD, right atrial dimension; RPLx, right parasternal long‐axis view; RPSx, right parasternal short‐axis view; RVDbase, basilar right ventricular dimension; RVWT, right ventricular wall thickness; SD, standard deviation.

Results of the Bland‐Altman analyses are summarized in Figure 2 and Table 2. For RAD, dimensions were significantly increased (fixed bias; *P* = .0001) when measured from the RPLx projection compared with the Ap4Ch view. This difference worsened as RA size decreased with significant proportional bias noted (*P* = .04). For basilar RV dimension, dimensions from the RPLx view were significantly decreased (fixed bias; *P* < .0001) compared with the Ap4Ch view. For RVWT, wall thickness was significantly increased from the RPLx view compared with the RPSx view (fixed bias; *P* = .0001). Limits of agreement were widest for the basilar RVD comparison and smallest for the RVWT comparison.

**TABLE 2:** Results of Bland‐Altman plot analyses evaluating agreement for equivalent right heart size measurements from different echocardiographic imaging planes in 90 healthy dogs.

Echocardiographic index (Method 1 − Method 2)	Mean difference (bias) as % (95% CI)	*P*‐value*	Lower limit (95% CI)	Upper limit (95% CI)	*P*‐value slope of regression line^#^
RA dimension (RAD_RPLx_/AoD − RAD_AP4Ch_/AoD)	7.8% (4.1, 11.5)	**.0001**	−26.1% (−32.3, −19.8)	41.7% (35.4, 47.9)	**.04**
Basilar RV dimension (RVDbase_RPLx_/AoD − RVDbase_AP4Ch_/AoD)	−14.9% (−19.0, −10.7)	**<.0001**	−53.5% (−60.6, −46.4)	23.7% (16.6, 30.8)	.51
RVWT (RVWT_RPLx_/AoD − RVWT_RPSx_/AoD)	4.9% (2.4, 7.3)	**.0001**	−17.8% (−22.0, −13.6)	27.6% (23.4, 31.7)	.42

*Note*: See Table 1 for the abbreviations key. *P*‐values that appear in bold type face denote statistical significance.

* Paired *t* test comparing percent difference of Method 1 and Method 2 where statistical significance suggests fixed bias.

^#^ Simple linear regression analysis where statistical significance suggests proportional bias.

Intraoperator (inter‐day) and interoperator (intra‐day) reproducibility of the right heart ratios are shown in Table 3. For intraoperator reproducibility, all CVs were ≤17.5%. Coefficients of variation were comparatively higher for interoperator reproducibility ranging from 7.5% to 22.6%. Like CVs, 95% RCs were higher for interoperator reproducibility compared with intraoperator reproducibility (with some exceptions).

**TABLE 3:** Reproducibility of right heart ratios from healthy 8 dogs that were examined on 3 separate days by the same operator and by 3 different operators.

Echocardiographic variable	Intraoperator (inter‐day) CV (%)	Intraoperator (inter‐day) 95% RC^a^	Interoperator CV (%)	Interoperator 95% RC^a^
Right atrial size				
RAD_RPLx_/AoD	6.1	0.28	8.6	0.39
RAD_AP4Ch_/AoD	7.8	0.30	7.5	0.29
RAD_RPLx_/LAD_RPLx_	9.0	0.12	14.0	0.18
RAD_AP4Ch_/LAD_AP4Ch_	8.6	0.20	7.9	0.18
Right ventricular chamber size				
RVDbase_RPLx_/AoD	7.6	0.26	13.4	0.41
RVD_RPSx_/AoD	14.3	0.39	17.9	0.46
RVDbase_AP4Ch_/AoD	7.3	0.26	8.9	0.30
RVDmid_AP4Ch_/AoD	7.7	0.21	13.1	0.38
RVDbase_RPLx_/LVDbase_RPLx_	9.6	0.16	16.4	0.24
RVD_RPSx_/LVD_RPSx_	14.7	0.19	20.7	0.25
RVDbase_AP4Ch_/LVDbase_AP4Ch_	9.4	0.17	9.0	0.16
Right ventricular wall thickness				
RVWT_RPLx_/AoD	12.8	0.15	15.8	0.19
RVWT_RPSx_/AoD	13.6	0.11	14.5	0.12
RVWT_RPLx_/LVWT_RPLx_	17.5	0.30	22.6	0.39
RVWT_RPSx_/LVWT_RPSx_	15.0	0.24	14.0	0.21
Other				
PVdia_RPSx_/AoD	4.2	0.12	8.9	0.25
EId	6.6	0.21	8.0	0.25
TAPSE/AoD	14.6	0.30	22.3	0.49

Abbreviations: 95% RC, 95% reproducibility coefficient; CV, coefficient of variation. See Table 1 for the rest of the abbreviations key.

^a^ 95% RC is in the same unit as the echocardiographic variable.

Results of measurement variability analyses that evaluated the investigator who performed the echocardiographic measurements for study purposes are presented in Table 4.

**TABLE 4:** Measurement variability of echocardiographic measurements involved in the current study from 8 randomly selected healthy dogs that were re‐measured on 2 different occasions and by 2 additional investigators.

Echocardiographic measurement	Intraobserver CV (%)	Interobserver CV (%)
RAD_RPLx_	3.8	12.9
RAD_AP4Ch_	4.7	8.2
RVDbase_RPLx_	7.0	13.1
RVD_RPSx_	11.3	11.5
RVDbase_AP4Ch_	9.2	12.7
RVDmid_AP4Ch_	10.6	15.5
RVWT_RPLx_	11.1	11.9
RVWT_RPSx_	15.7	15.8
PVdia_RPSx_	4.5	5.6
AoD	3.4	5.9
LAD_RPLx_	4.6	8.8
LAD_AP4Ch_	6.1	8.7
LVDbase_RPLx_	4.3	4.2
LVD_RPSx_	2.0	3.2
LVD1_RPSx_	5.3	10.1
LVDbase_AP4Ch_	4.9	6.3
LVWT_RPLx_	12.2	12.3
LVWT_RPSx_	8.9	11.3
TAPSE	9.0	17.3

Abbreviations: CV, coefficient of variation; LVD1, latero‐lateral left ventricular dimension. See Table 1 for the rest of the abbreviations key.

## 4 DISCUSSION

We describe simple linear 2‐dimensional echocardiographic methods for identifying abnormalities of right heart size and function in dogs. The study provides reference intervals derived from a relatively large and diverse sample of dogs for numerous body weight‐independent indices of right heart size and function, which are indexed to the long‐axis aortic diameter or the corresponding left heart structure (right heart ratios). These right heart ratios are convenient because they negate the need to normalize the right heart measurement to body weight, which requires knowledge of a scaling exponent or access to a chart with prediction intervals based on arbitrarily defined body weight values. Selected method comparison of RAD and RVDbase from different echocardiographic views (RPLx and Ap4Ch) suggests that measurements are not interchangeable. Measurements of RVWT seem interchangeable when measured from RPLx and RPSx. We also evaluated the variability of right heart ratios that results from repeated echocardiographic examinations on different days and by different operators. The reported 95% RCs are useful to detect progression or regression of abnormalities during reevaluations.

Despite the quantitative echocardiographic assessment of the canine right heart becoming increasingly important,^1^, ^2^, ^3^, ^4^, ^5^, ^6^, ^7^, ^8^, ^9^, ^10^, ^11^, ^12^, ^13^, ^14^ measurements of right heart size and function for clinical purposes seem to be performed on a limited basis, even in patients with right heart disease. Thus, simple and convenient body weight‐independent methods for right heart assessment may be useful to facilitate quantitative assessment for routine clinical practice. Many clinicians do not have access to or time for advanced imaging of the right heart such as strain imaging or 3‐dimensional echocardiography despite their advantages. Similar to left heart ratios,^25^, ^26^, ^27^, ^28^, ^29^ right heart ratios offer a convenient technique to assess right heart size and function.^18^ A unique aspect of our study is that we indexed right heart measurements to the long‐axis AoD and not the short‐axis aortic root. This approach was intended to avoid the aforementioned challenges and potential imprecision associated with the short‐axis aortic root. The AoD measurement also is our preference when indexing linear measurements to the aorta.

We also report right heart size measurements compared with the equivalent left heart structure. Many sonographers evaluate right heart size subjectively and relative to the corresponding left heart structure, especially in the setting of focused or emergency point‐of‐care cardiac ultrasound examination.^41^, ^42^, ^43^, ^44^, ^45^ Quantitative linear measurements of LA and left ventricular size are encouraged, likely because quantitative right heart measurements have lagged behind left heart measurements. Common anecdotal recommendations to assess right heart size state that, from the RPLx or Ap4Ch views, the RA should be not be larger in size relative to the LA, the RV minor chamber dimension is commonly said to be no larger and usually <50% of the left ventricle, and RV wall thickness should be one‐half to one‐third the thickness of the left ventricle. We are unaware of studies verifying these recommendations with the exception of a study that evaluated RV/left ventricular minor chamber dimension ratio from an RPLx 5‐chamber view using an M‐mode transducer in 25 dogs in which values ranged from 0.27 to 0.5.^46^ Results of our study mostly validate previous anecdotal recommendations. The upper reference limit for RAD/LAD was 0.95 from the RPLx view and 1.07 from the Ap4Ch view. The upper reference limit for RVD/LVD was 0.68 from the RPLx view, 0.54 from the RPSx view, and 0.76 from the Ap4Ch view. The upper reference limit for RVWT/LVWT was 0.78 from the RPLx view and 0.77 from RPSx, which is larger than the aforementioned recommendation. Indeed, even median values for RVWT were >0.5.

We elected to report both types of right heart ratios (right heart measurements indexed to the corresponding left heart structure and AoD) in our study to provide options for right heart size assessment. However, caution is advised when using right heart measurements indexed to the corresponding left heart structure. These right heart ratios are unreliable in the commonly encountered contexts of left heart abnormalities or disease. Right heart measurements indexed to AoD would be a better choice in most situations. Aortic disease or size abnormalities are much less common as compared with changes (increase or decrease) in left heart size.

When comparing equivalent measurements of right heart size from different echocardiographic views, consistent differences were identified based on the evidence of fixed bias for comparisons of RAD, RVDbase, and RVWT. However, differences and limits of agreement were largest for comparisons of RAD and especially RVDbase. This observation suggests these measurements should not be considered interchangeable in most dogs and should be measured consistently from the same echocardiographic view (ie, RPLx or Ap4Ch) during serial examinations. This factor seems to be of lesser importance when assessing RVWT, because differences in measurements from RPLx and RPSx were small with acceptable limits of agreement.

Our measurement PVdia_RPSx_/AoD differs from a previously reported measurement of pulmonary valve size in that we performed the measurement during early to midsystole (versus early diastole) and indexed it to the equivalent AoD from a RPLx view and not the aortic root from a RPSx basilar view.^15^ Comparing PVdia_RPSx_ size to aortic root size from the RPSx basilar view will yield smaller ratios, in as much as this aortic measurement is larger than AoD because it includes measurement of at least 1 sinus of Valsalva. Indeed, the range of values observed in the previous study was 0.70 to 0.98 compared with 0.76 to 1.53 in ours. Similarly, a previous study proposed reference intervals for TAPSE/Ao in 50 healthy dogs,^18^ but differs from our measurement of TAPSE/AoD for similar reasons related to differences in how the aortic diameter was measured. Finally, EId recently was evaluated in a study of dogs with pulmonary hypertension,^36^ including in a 29 healthy control dogs. Median (minimum‐maximum) values were similar 1.12 (0.94‐1.30) when compared with our results 1.04 (0.81‐1.32).

Comparing the reproducibility of the right heart ratios determined in our study compared with other studies that assessed reproducibility of right heart measurements should be avoided. Coefficients of variation are by definition highly dependent on the study population mean specific to that measurement or index. They are not generally applicable to compare across studies.^39^ However, comparisons of different indices within our study can be made and prompts 2 general observations. First and unsurprisingly, intraoperator CVs were lower compared with interoperator CVs, suggesting serial measurements performed by the same operator have lower measurement variability. Also, right heart measurements indexed to AoD had slightly lower CVs compared with right heart measurements indexed to the corresponding left heart structure. This finding suggests that the right heart measurements indexed to AoD carry less measurement variability compared with the analogous right heart measurement indexed to the corresponding left heart structure. The 95% RC values reported are more generalizable and have clinical relevance. These values describe a minimum detectable difference that helps distinguish (with 95% confidence) a real change during serial evaluations from change caused by measurement variability.^40^ For example, our study suggests that during repeat examination a meaningful increase in RA size using RAD_RPLx_/AoD would be an increase of at least 0.28 (eg, an increase from 1.0 to 1.28) when assessed by the same sonographer and an increase of at least 0.39 (eg, an increase from 1.0 to 1.39) when assessed by a different sonographer.

We performed a quality control assessment of the investigator who performed the measurements for study purposes (Table 4). This approach was used primarily because the investigator (JNS) was not an experienced cardiologist but a recently trained investigator. This assessment was intended to evaluate the consistency of the investigator's measurements when repeated on separate occasions and when evaluated against the other investigators, including an experienced cardiologist. These results show all CVs were <18% and all but 4 CVs <15%, suggesting adequate training and consistency of measurements performed by this investigator for study purposes.

It should be noted that RVWT_RPLx_/AoD, RVWT_RPSx_/AoD, and TAPSE/AoD were not completely independent of body weight. Weak but significant negative associations with body weight were identified. The reason for these associations is unclear other than the possibility that changes in AoD with body size are not proportional to changes in RVWT and TAPSE. A previous study also identified a weak but significant negative association between TAPSE and aortic root diameter from the RPSx basilar view.^18^

Our results should be viewed within the context of the study's limitations. Our study design was cross‐sectional, included a limited number of dogs, and included 1 sonographer and 1 individual who performed echocardiographic measurements. Thus, we cannot be certain that all dogs included were free of disease and our results are not representative of all dogs and all sonographers. Thus, the obtained reference intervals should help guide clinical decision‐making, but should not be the sole basis for clinical decisions. Right heart ratios inherently rely on the assumption that AoD or the corresponding left heart structure is normal, which may not be the case in some clinical scenarios. For example, these results are not applicable to dogs with left ventricular outflow tract or aortic abnormalities (eg, subaortic/aortic stenosis). Right heart chamber size measurements performed in our study were particularly dependent on the imaging planes used. For example, experienced sonographers acknowledge that right heart chamber size from the Ap4Ch view can change markedly depending on the angle of insonation and specific imagining techniques used. Thus, our reference intervals are specific to the acquisition techniques described in the study.

In conclusion, reference intervals for simple body weight‐independent right heart ratios (ie, right heart measurements indexed to the AoD or corresponding left heart structure) for quantitative evaluation of right heart size and function are available for clinical use. Reproducibility assessment of the right heart ratios provides potentially useful information that can help guide interpretation of changes in right heart size observed during serial evaluations.

## CONFLICT OF INTEREST DECLARATION

Authors declare no conflict of interest.

## OFF‐LABEL ANTIMICROBIAL DECLARATION

Authors declare no off‐label use of antimicrobials.

## INSTITUTIONAL ANIMAL CARE AND USE COMMITTEE (IACUC) OR OTHER APPROVAL DECLARATION

Approved by the IACUC at Colorado State University (protocol #4429).

## HUMAN ETHICS APPROVAL DECLARATION

Authors declare human ethics approval was not needed for this study.
